# Ionomic Profile of Rice Seedlings after Foliar Application of Selenium Nanoparticles

**DOI:** 10.3390/toxics12070482

**Published:** 2024-07-01

**Authors:** Bruna Moreira Freire, Camila Neves Lange, Yasmin Tavares Cavalcanti, Amedea Barozzi Seabra, Bruno Lemos Batista

**Affiliations:** 1 Center for Natural and Human Sciences (CCNH), Federal University of ABC (UFABC), Santo André 09210-580, SP, Brazil; 2 Department of Analytical Chemistry, Aragon Institute of Engineering Research (I3A), University of Zaragoza, 50009 Zaragoza, Spain; 3 National Institute of Science and Technology in Nanotechnology for Sustainable Agriculture, INCTNanoAgro, Santo André 09210-580, SP, Brazil

**Keywords:** SeNPs, sodium selenite, *Oryza sativa* L., agronomic biofortification, transfer factor, arsenic, lead

## Abstract

Nanotechnology has been increasingly used in plant sciences, with engineered nanoparticles showing promising results as fertilizers or pesticides. The present study compared the effects in the foliar application of Se nanoparticles (SeNPs) or sodium selenite-Se(IV) on rice seedlings. The degree of plant growth, photosynthetic pigment content, and concentrations of Se, Na, Mg, K, Ca, Mn, Co, Cu, Zn, As, Cd, and Pb were evaluated. The results showed that the application of SeNPs at high concentrations (5 mg L^−1^), as well as the application of Se(IV), inhibited plant growth and increased the root concentrations of As and Pb. The application of SeNPs at 0.5 mg L^−1^ significantly increased Se accumulation in the aerial part from 0.161 ± 0.028 mg kg^−1^ to 0.836 ± 0.097 mg kg^−1^ without influencing physiological, chemical, or biochemical parameters. When applied to leaves, SeNPs tended to remain in the aerial part, while the application of Se(IV) caused a higher Se translocation from the shoots to the roots. This study provides useful information concerning the uptake, accumulation, and translocation of different Se formulations in rice seedlings and their effect on plant ionomic profiles, thus showing that the foliar application of SeNPs at low concentrations can be an effective and safe alternative for rice biofortification.

## 1. Introduction

The exponential growth of the world population has led to an increasing demand for food, causing an exhaustive use of soil. Some consequences are the loss of soil fertility and the search for new fertilizers and pesticides to increase productivity and suppress crop diseases [1,2]. According to FAO [3], currently, more than 820 million people are hungry, mainly in Africa, Latin America, and Western Asia. Meanwhile, another 2 billion people worldwide experience food insecurity. In this sense, new strategies for sustainable agriculture to combat hunger and malnutrition while ensuring food security and safety are necessary [3].

In recent decades, nanotechnology has driven a revolution in plant science studies [4]. Engineering nanoparticles (NPs) have been gaining popularity as a safer and more effective alternative to conventional fertilizers and pesticides [2,5], thus contributing to enhancing food production and security. The use of NPs during plant cultivation may enhance crop growth and productivity, stimulate the antioxidant system, mitigate reactive oxygen species, and alleviate the undesirable effects of the accumulation of potentially toxic elements (PTEs) [2,6,7,8,9]. However, one of the objectives of significant global interest related to the application of NPs to crop cultivation is promoting agronomic biofortification, that is, increasing the nutritional value of a food crop [10,11].

Selenium (Se) is an essential micronutrient for humans, and a deficiency of it can lead to the development of some diseases, such as hypothyroidism and heart disease [12]. The recommended dietary intake of Se is 55 µg day^−1^ [13]. However, it is estimated that one billion people have insufficient Se intake [14]. Plants are the main source of Se for humans [15], but intensive agriculture contributes to Se becoming increasingly rare in foods [16]. Agronomic biofortification using Se compounds as fertilizers is an effective strategy to increase the Se content in edible crops and then ensure that food products will have the adequate amounts of Se required for humans [15,16]. Currently, Se biofortification is mainly based on ionic formulations, such as sodium selenite- Se(IV) or sodium selenate- Se(VI) [11,17]. Indeed, in their nanoformulation, selenium nanoparticles (SeNPs) have drawn considerable attention for agricultural purposes. Several studies have reported the benefits of SeNPs for plants [6,11,18]. However, it is worth mentioning that SeNPs have a dual effect on plants, that is, depending on the concentration, they can act as plant stimulators or inhibitors [6]. Agronomic biofortification with SeNPs can be achieved by foliar spray or soil irrigation, but the first is preferred and more effective [11]. Rice is among the most consumed cereals in the human diet, making it one of the most suitable targets for agronomic biofortification [15].

Despite the promising results of nanotechnology being applied to agriculture, further research is needed to better understand NP–plant interactions. According to Freitas et al. [4], multiple parameters need to be taken into consideration to evaluate the beneficial effects or toxicity of NPs on plants, such as visual indicators and the quantification of compounds within parts of the plant. The ionomic profile of edible plants is an important parameter to be studied as the application of SeNPs could influence the human nutrient intake and exposure to PTEs. In *Raphanus sativus* and *Brassica juncea* plants, SeNPs had a negative impact on the uptake of Zn [10]. Se(IV) and Se (VI) affected the macro- and micronutrient contents of rice grains [19]. Studies have highlighted the potential of both Se(IV) and SeNPs in mitigating As toxicity in rice seedlings by reducing the As translocation from roots to shoots [20,21]. SeNPs also decreased Cd accumulation in rice cultivated in Pb-and-Cd-combined paddy soils [22]. However, these studies generally (1) relied on hydroponic experiments, which do not accurately reflect the conditions of rice cultivation; (2) did not compare the effects of Se in ionic and nanoparticle form; or (3) evaluated the impact of SeNPs on only one or a few elements, thus not studying the broader ionomic profiles of the nutrients and PTEs.

Therefore, we hypothesized that different Se formulations and concentrations applied via foliar spray in pot experiments would affect the growth, chemical element composition, and photosynthetic pigment content of rice plants (*Oryza sativa* L.). The main objectives of this study were the following: (1) to compare the effects of SeNPs and Se(IV) on rice seedling growth; (2) to assess the influence of Se applications on photosynthetic pigments in rice leaves; (3) to elucidate the foliar uptake and translocation of Se in rice seedlings and the potential of the different treatments for agronomic biofortification; and (4) to evaluate the influence of the treatments on macronutrient, micronutrient, and PTE uptake and accumulation.

## 2. Materials and Methods

### 2.1. SeNP Synthesis and Characterization

In this report, selenite (Na_2_SeO_3_) and selenate (Na_2_SeO_4_) are abbreviated as Se(IV) and Se(VI), respectively. SeNPs were synthesized, in accordance with Freire et al. [23], by a chemical reduction in the Se(IV) (Sigma-Aldrich, St. Louis, MO, USA) by ascorbic acid (Synth, Diadema, SP, Brazil) using polyvinyl alcohol (PVA 30,000 to 70,000 MW, Sigma-Aldrich, St. Louis, MO, USA) as a stabilizer. The synthesized SeNPs were previously characterized by ultraviolet-visible spectrophotometry (UV-vis, Agilent 8454, Palo Alto, CA, USA) and transmission electron microscopy (TEM, JEM-2100 Plus, 200 kV, JEOL, Peabody, MA, USA) [23].

To estimate the formation efficiency of SeNPs, a suspension of SeNPs (the theoretical calculated Se concentration: 801 mg L^−1^) was prepared, transferred to Amicon Ultra-4 10 kDa centrifugal filter tubes (maximum pore size ~3 nm, Millipore, Ireland), and then centrifuged at 13,400 rpm for 60 min. The filtrate was oxidized with 2.5% *v*/*v* HNO_3_, analyzed (n = 3) for total Se content by inductively coupled plasma mass spectrometry (ICP-MS, Agilent 7900, Hachioji, TY, Japan), and then considered as the ionic fraction. The original synthesized suspension was acid-digested (n = 3), in accordance with Freire et al. [23], analyzed by ICP-MS, and then considered as 100% of Se. Briefly, 0.5 mL of SeNPs was digested with 1 mL of sub-distilled nitric acid (HNO_3_ 65%, Synth, Diadema, SP, Brazil) at room temperature for 2 h. The final solution was 10,000-fold diluted.

Finally, the SeNPs were diluted to 400 mg L^−1^ in ultrapure water or in the medium used to perform foliar application (0.1% m/v Triton X-100, Sigma-Aldrich, USA), and they were then characterized by dynamic light scattering (DLS, Nano ZS Zetasizer, Malvern Instruments Co., Worcestershire, UK) to compare the hydrodynamic diameter, size distribution, polydispersity index (PdI), and zeta potential in both media.

### 2.2. Soil Characterization

The soil used in the pot experiments was characterized by its general physicochemical properties following the procedures described by da Silva [24] and Raij et al. [25]. The texture was classified as clay soil, with a moderate-to-slightly acidic pH. In addition, several chemical elements were quantified in the soil (Se, Co, Cu, Zn, As, Cd, and Pb) by ICP-MS. For this purpose, 150 mg of soil (n = 3) was pre-digested overnight with 3 mL of concentrated HNO_3_. Then, the samples were heated in a digesting block at 95 °C for 4 h and diluted to 50 mL with ultrapure water. Table 1 shows the results of the soil characterization. The soil was rich in organic matter and free from any PTE contamination.

### 2.3. Plant Material and Growth Conditions

Rice seeds (*Oryza sativa* L. ssp. *indica* cv. BRS PAMPA) were provided by Brazilian Agricultural Research Corporation (EMBRAPA, Temperate Climate Center, Pelotas, RS, Brazil) and stored in the dark in dry conditions before their use in order to avoid loss of their viability [26]. The pot trial was carried out in the greenhouse of the Federal University of ABC (UFABC, São Bernardo do Campo, Brazil) from September to October 2021.

The *O. sativa* seeds were soaked in water in the dark for 48 h. Then, the seeds were germinated on paper sheets moistened with water at 28 °C (SL-100, Solab, Brazil). After 72 h, the uniform seedlings were selected and transferred to 200 mL plastic pots (one seedling per pot), which contained the soil previously characterized. The water regime was flooding, with 3–4 cm of water after 10 d until the end of the experiment. At 25 d, the seedlings were transferred to 590 mL plastic pots. They remained there until the end of the vegetative period (around 45 d). Plants were grown at 30 ± 5 °C and in a relative humidity between 60 and 75%.

### 2.4. Plant Treatments

The experimental design was a completely randomized 3 × 2 factorial scheme with three Se concentrations (0, 0.5, and 5.0 mg L^−1^) and two Se sources (the synthesized SeNPs and Se(IV)). The applied Se concentrations were selected based on a previous study from our group [6], which revealed that the application of SeNPs at 0.5 mg L^−1^ showed beneficial effects on rice germinating seeds.

The foliar spray applications were performed at 20, 23, 40, and 43 d (the frequency of the applications was chosen via thinking of the compound in a real crop, i.e., the number of applications was not exaggerated in trying to improve the possibility of absorption by the plant). Each group (control, 0.5 mg L^−1^ SeNPs, 5 mg L^−1^ SeNPs, 0.5 mg L^−1^ Se(IV), and 5 mg L^−1^Se(IV)) consisted of 10 biological replicates. A total of 50 pots were used for this experiment. A 0.1% m/v solution of Triton X-100 was used as a surfactant for all dilutions. Control plants were sprayed with this 0.1% Triton X-100 solution. The SeNP suspensions were freshly diluted after sonication of the synthesized stock suspension for 1 min (KQ3200, 120 W, 40 KHz, Kunshan, China). To prevent the SeNPs from leaching into the soil, the soil surface was covered with aluminum foil during foliar applications [27]. Solutions of Triton X-100, Se(IV), or suspensions of SeNPs were sprayed onto the plant leaves until the solution was homogeneously distributed on the leaf’s surface [19]. All applications were carried out in the morning, i.e., between 6:30 and 7:30.

### 2.5. Growth Measurements

At 45 d of seedling growth, the agronomic parameters were measured for six replicates per treatment group. Plant height was measured using a ruler, and the number of leaves and tillers was counted. Then, plant tissues (roots and aerial parts, consisting of shoots and leaves) were collected, separated, washed under running water, and rinsed with deionized water. Fresh leaf samples from each group were separated and stored at −80 °C for the determination of photosynthetic pigments, as described in Section 2.6. Roots, shoots, and leaves were oven-dried at 45 °C to a constant mass, weighed, and roughly cut into small pieces for acid digestion, as described in Section 2.7.

### 2.6. Carotenoid and Chlorophyll Analyses

The determination of the chlorophyll a (C_a_), chlorophyll b (C_b_), and carotenoids (Car) was carried out by UV-vis spectrophotometry (Agilent, model 8453, Palo Alto, CA, USA). About 50 mg of fresh rice leaves were macerated with a mortar and pestle in ethanol (99% *w*/*v*, LabSynth, Diadema, SP, Brazil). The extract was made up to 10 mL with ethanol in test tubes and stored at 4 °C in the dark. After 96 h, the absorbance readings were recorded at the wavelengths of 470, 649, and 665 nm. Six replicates were analyzed per group. Concentrations of C_a_, C_b,_ and Car (mg g^−1^ FW) were calculated according to Lichtenthaler and Wellburn [28], following Equations (1)–(3):

C_a_ = (13.95 × A_665_) − (6.88 × A_649_), (1)

C_b_ = (24.96 × A_649_) − (7.32 × A_665_), (2)

Car = ((1000 × A_470_) − (2.05 × C_a_) − (114.8 × C_b_))/245, (3)

where A_665_, A_649_, and A_470_ are the absorbances at 665, 649, and 470 nm, respectively.

### 2.7. Quantification of the Nutritional and Potentially Toxic Elements by ICP-MS

According to Boldrin et al. [19], the application of Se may affect the macro- and micronutrient contents of rice. Therefore, the total concentrations of Se and other elements (Na, Mg, K, Ca, Mn, Co, Cu, Zn, As, Cd, and Pb) were determined in the acid-digested roots and leaves.

Plant sample preparation and analyses for elemental determination were based on Paniz et al. [29], with minor modifications. Briefly, portions of 50 mg (for roots) or 100 mg (for leaves) of ground samples were weighed (six replicates per group), and HNO_3_ 65% was added (1 mL for roots and 1.5 mL for leaves). After 48 h of pre-digestion at room temperature, the samples were heated at 95 °C in a digester block (EasyDigest, Analab, Wantzenau, France) for 4 h. After cooling, the volume was made up to 20 mL with ultrapure water, and the samples were analyzed by ICP-MS. Analytical blanks were prepared and analyzed throughout the experiments.

To minimize spectral interferences, He and H_2_ (99.999%, White Martins, Mauá, SP, Brazil) were used in the collision reaction cell (CRC). The ICP-MS instrument operating conditions are summarized in the Supplementary Materials (Table S1). Calibration curves in the range of 0–200 µg L^−1^ (Mn, Co, Cu, Zn, As, Se, Cd, and Pb) and 0–5000 µg L^−1^ (Na, Mg, K, and Ca) were prepared by dilutions of the multi-element standard solutions (10 µg mL^−1^, Perkin Elmer, Norwalk, CT, USA). The limits of detection (LoD) and quantification (LoQ) were calculated, in accordance with the National Institute of Metrology, Quality, and Technology [30], as the mean of the analytical blanks’ concentration values added with three (LoD) or ten times (LoQ) the standard deviation of these measurements (n = 10).

#### 2.7.1. Quality Control

Five standard reference materials (SRMs) were used to evaluate the accuracy of the method: natural water (NIST 1640a, National Institute of Standard and Technology, Gaithersburg, MD, USA); tomato leaves (NIST 1573a, Gaithersburg, MD, USA); tomato leaves (C1003a, CRM-Agro—Certified Reference Materials for Agriculture, Livestock and Toxicology, CENA-USP, Piracicaba, SP, Brazil); aquatic plants (BCR-670, Institute for Reference Materials and Measurements, Geel, Belgium); and sandy clay soil (CRM049, Sigma-Aldrich, Laramie, WY, USA). The SRMs were prepared following the same procedure described for sample preparation in Section 2.2 and Section 2.7. A minimum of three replicates were analyzed, and the results obtained, as well as certified values and recoveries, are in the Supplementary Materials (Table S2). The recoveries of all the elements were in the range of 79 to 119%, except for Co in SRM C1003a, where the recovery was lower (64%); however, the obtained result (211 ± 7 µg kg^−1^) was within the SRM uncertainty range (330 ± 140 µg kg^−1^).

#### 2.7.2. Data Analysis

The element concentrations in the roots and leaves were calculated on a dry weight (DW) basis. The total element accumulation in the roots and leaves (T_root_ and T_leaf_) were calculated following Equations (4) and (5):

T_root_ = C_root_ × Root_drybiomass._ (4)

T_leaf_ = C_leaf_ × Leaf_drybiomass._ (5)

The transfer factors (TFs) from the roots to the aerial parts were calculated for Se, As, Cd, and Pb by dividing the element concentration in the leaves (C_leaf_) by the concentration in the roots (C_root_), as per Equation (6).

TF = C_leaf_/C_root._ (6)

### 2.8. Statistical Analysis

Significant differences (at a significance level of 0.05) were evaluated by one-way analysis of variance (ANOVA). All statistical analyses were performed using Statistica software (v.7.0, StatSoft, Tulsa, OK, USA) and GraphPad Prism 10.2.2 (GraphPad Software, San Diego, CA, USA).

## 3. Results and Discussion

### 3.1. SeNP Characterization

The characterization of SeNPs by UV-vis and TEM has been reported elsewhere [23], where the synthesis of spherical and monodisperse SeNPs with an average diameter of 50.1 ± 5.6 nm was confirmed. A representative TEM image of synthesized SeNPs is shown in Supplementary Materials Figure S1.

The Se content determined in the original synthesized SeNPs suspension was 787 ± 9 mg L^−1^, while the ionic fraction was 195 ± 13 mg L^−1^, resulting in 75% of SeNP formation efficiency. Figure S2 shows the size distributions obtained by DLS for the SeNPs diluted in ultrapure water (red line) or in 0.1% Triton X-100 (green line). The mean hydrodynamic sizes obtained for both dilutions (95 ± 20 and 72 ± 17 nm, respectively) showed no statistical difference at the 95% significance level (*p* > 0.05). The size distributions (Figure S2) showed only a small shift to smaller sizes after dilution with Triton X-100, with results closer to the average diameter obtained by TEM (50.1 nm), thus indicating that the use of surfactants may improve the dispersity of SeNPs. The PdIs obtained for dilutions in water and in Triton X-100 were 0.009 ± 0.006 and 0.010 ± 0.005, respectively, thus confirming monodispersity in both cases. The zeta potential for the Triton X-100 dilution (−9.85 ± 0.39 mV) was higher in module than that obtained for water dilution (−3.63 ± 2.58), thus confirming that the use of surfactants improves SeNPs stability.

### 3.2. Effect of Se on Rice Seedling Growth

**Figure 1:**
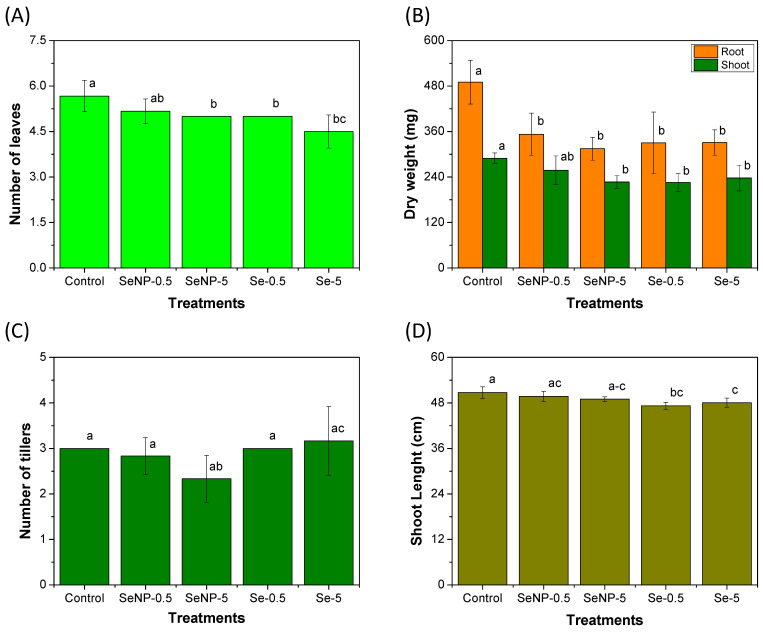
The growth parameters measured in the rice seedlings after 45 days of cultivation when under foliar application of the treatments. (**A**) Number of leaves; (**B**) root and shoot dry weight; (**C**) number of tillers; and (**D**) shoot length. The data presented are the means of six replicates, while the error bar represents the standard deviation. Different letters represent a significant difference between the treatments (*p* < 0.05) for each variable. The treatment groups were as follows: control; SeNP−0.5: application of selenium nanoparticles at a concentration of 0.5 mg L^−1^ of Se; SeNP−5: application of selenium nanoparticles at a concentration of 5.0 mg L^−1^ of Se; Se−0.5: application of sodium selenite at a concentration of 0.5 mg L^−1^ of Se; and Se−5: application of sodium selenite at a concentration of 5.0 mg L^−1^ of Se.

A previous study conducted by our group showed that the application of SeNPs at 0.5 mg L^−1^ in germinating rice seeds stimulated the antioxidant system without affecting seedling growth [6]. Therefore, in the present study, we evaluated the foliar application of SeNPs in rice seedlings at the optimal concentration selected in the study mentioned above and at a concentration that was 10-fold higher (5.0 mg L^−1^). Solutions of Se(IV) at the same Se concentrations were also applied to compare the effects observed when Se is in ionic or nanoparticulate form. Supplementary Materials Figure S3 shows representative images of the rice plants under different treatments after 45 days of cultivation. Visually, no toxicity effects were observed on the plants, such as chlorosis spots or a visual decrease in plant height. However, the quantitative results of the growth parameters measured on rice seedlings at the end of the experiment were detailed, as shown in Figure 1.

Significant differences (*p* < 0.05) between the groups were observed for all evaluated parameters (number of leaves, roots and shoots dry weight, number of tillers, and shoot length). A tendency to decrease the growth parameters for Se-treated plants compared to the control plants was observed in Figure 1A,B. The highest concentration of SeNPs (5 mg L^−1^), as well as the two concentrations of Se(IV) (0.5 and 5 mg L^−1^), inhibited plant growth, as evidenced by the decreased number of leaves and shoot dry weight. All treatments decreased the root’s dry weight compared to the control group, and no statistical differences (*p* < 0.05) between the Se treatments were observed for this parameter (Figure 1B). The application of Se(IV) at the 5 mg L^−1^ level produced plants with a higher number of tillers compared to the application of SeNPs at the same concentration (Figure 1C), but none of the groups were statistically different from the control. Finally, the application of Se(IV), regardless of the concentration, decreased the shoot length compared to the control (Figure 1D). Nonetheless, the administration of SeNPs at 0.5 mg L^−1^ only affected the root’s dry mass compared to the control (Figure 1B). The higher toxicity of SeNPs to rice roots compared to other tissues has previously been reported [6].

The results of the present study suggest that the application of SeNPs in high concentrations, as well as the application of Se(IV), can negatively influence the physiological characteristics of *O. sativa*, while the application of SeNPs at low concentrations (0.5 mg L^−1^) show no signs of toxicity. In agreement with our results, Fidelis et al. [31] observed a higher phytotoxic effect of Se(IV) compared to elemental Se on rice seedlings, as evidenced by the inhibition in germination and seedling development. This difference in toxicity can be justified by the different modes of Se absorption. Se(IV) is quickly absorbed and translocated to plant tissues, where it can be converted into organic Se and replace S in proteins, thus causing toxicity [31,32].

Different impacts of Se in nanoparticulate or ionic form in plants have been reported. Neysanian et al. [33] observed that the foliar application of 3 mg L^−1^ of Se as Se(VI) or SeNPs in tomato plants increased root and shoot biomass and fruit production. On the other hand, similar to our results, the application of Se in higher concentrations (10 mg L^−1^), especially in the ionic form, induces toxicity [33]. Other studies have reported a dose-dependent phytotoxicity of Se in the ionic [31] or nanoparticulate form [6] to rice seedlings. Many factors can be related to the effects of SeNP application, such as NP size, surface chemistry, plant type, application form (foliar or by irrigation), concentration, and the number of applications, as reported by Pelegrino et al. [8], who applied CuO NPs to lettuce plants.

### 3.3. Effect of Se on Photosynthetic Pigments

**Figure 2:**
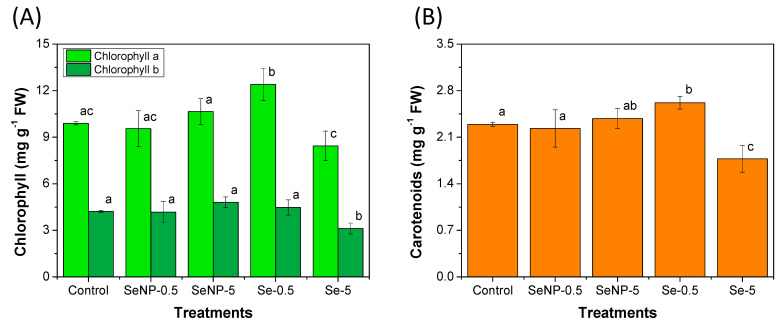
Concentrations of (**A**) chlorophyll a and b and (**B**) carotenoids measured in rice seedlings after 45 days of cultivation under foliar applications of the treatments. The data presented are the means of six replicates, while the error bar represents the standard deviation. Different letters represent a significant difference between the treatments (*p* < 0.05) for each variable. The treatment groups were as follows: control; SeNP−0.5: application of selenium nanoparticles at a concentration of 0.5 mg L^−1^ of Se; SeNP−5: application of selenium nanoparticles at a concentration of 5.0 mg L^−1^ of Se; Se−0.5: application of sodium selenite at a concentration of 0.5 mg L^−1^ of Se; and Se−5: application of sodium selenite at a concentration of 5.0 mg L^−1^ of Se.

Significant differences (*p* < 0.05) were observed for the Se(IV) treatments in concentrations of C_a_, C_b,_ and Car compared to the control group; meanwhile, for the SeNP treatments, no significant differences were observed (Figure 2). An increase in C_a_ content was noticed after the application of Se(IV) at 0.5 mg L^−1^, followed by a decrease at 5 mg L^−1^ (Figure 2A).

The application of 5 mg L^−1^ of Se(IV) also decreased the concentration of C_b_ (Figure 2A), while the other treatments did not significantly affect the levels of this pigment. Carotenoids followed the same behavior as C_a_, that is, an increase in its content was observed for the lower Se(IV) concentration (0.5 mg L^−1^), followed by a decrease for the highest Se(IV) concentration (5 mg L^−1^), as shown in Figure 2B.

The results indicate that the application of SeNPs did not significantly influence the photosynthetic pigments in the leaves of the rice seedlings. Nevertheless, an increasing trend, without statistical significance, was observed after the application of SeNPs at 5 mg L^−1^. On the other hand, the Se(IV) at low concentrations (0.5 mg L^−1^) increased in pigment content, while it decreased C_a_, C_b,_ and Car content at high concentrations (5 mg L^−1^). This may be related to the dose-dependent toxic effect of Se(IV) on plants. A possible reason is that the application of Se salt at high concentrations on the leaves may hamper the absorption of light, thus causing a decrease in photosynthetic pigments. In contrast, the spherical shape and the size at the nanometer scale of SeNPs did not prevent light absorption by the plant. The reduction in the number of leaves when Se(IV) was applied at 5 mg L^−1^ (Figure 1A) may also have contributed to the decreased production of photosynthetic pigments by this group.

In coffee plants, the foliar application of Se(VI) or SeNPs up to 40 mg L^−1^ increased the concentrations of C_a_, C_b,_ and Car. At higher concentrations (160 mg L^−1^), a decrease in the photosynthetic pigments was observed compared to the control plants, thus indicating a possible toxic effect [18].

### 3.4. Effect of Se on the Elemental Uptake and Accumulation in Rice Seedlings

#### 3.4.1. Selenium Accumulation

**Figure 3:**
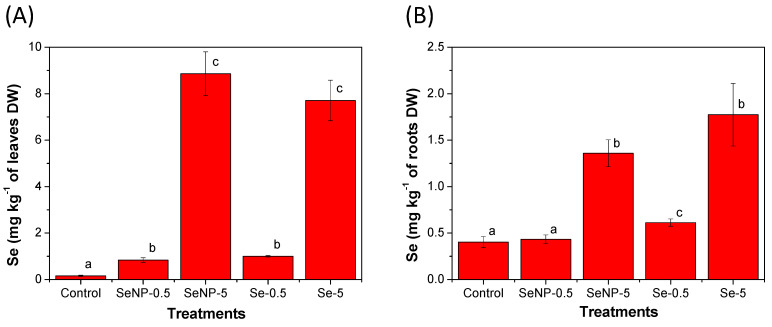
Selenium concentrations in the (**A**) leaves and (**B**) roots of rice seedlings after 45 days of cultivation under foliar applications of the treatments. The data presented are the means of six replicates, while the error bar represents the standard deviation. Different letters represent a significant difference between the treatments (*p* < 0.05) for each variable. The treatment groups were as follows: control; SeNP−0.5: application of selenium nanoparticles at a concentration of 0.5 mg L^−1^ of Se; SeNP−5: application of selenium nanoparticles at a concentration of 5.0 mg L^−1^ of Se; Se−0.5: application of sodium selenite at a concentration of 0.5 mg L^−1^ of Se; and Se−5: application of sodium selenite at a concentration of 5.0 mg L^−1^ of Se.

The application of Se, whether as SeNPs or Se(IV), increased the Se concentrations in the rice roots and leaves in a dose-dependent manner, as can be seen in Figure 3. The average Se concentration in the leaves (Figure 3A) increased from 0.161 ± 0.028 mg kg ^−1^ in the control group to 0.836 ± 0.097 mg kg^−1^ following SeNP application at 0.5 mg L^−1^, which is an increase of 420%. The application of Se(IV) at 0.5 mg L^−1^ increased leaf Se concentration by 520% compared to the control group, reaching 1.00 ± 0.036 mg kg^−1^. The foliar application of SeNPs or Se(IV) at 5 mg L^−1^ significantly increased Se concentrations in both leaves and roots compared to other treatment groups. The T_Se_ accumulation in roots and leaves also increased in the same groups (Supplementary Materials Table S3), thus confirming Se uptake. In the rice leaves, the Se concentrations following 5 mg L^−1^ SeNP or Se(IV) applications were remarkably higher than in the control group (55- and 48-fold, respectively). Our findings agree with Reis et al. [34], who reported that rice leaf Se content is directly affected by Se application. The applied Se can be absorbed by rice leaves through the cuticle or via the stomata.

Reis et al. [34] reported that the soil Se(VI) application at 25 g ha^−1^ increased leaf Se concentration in another Brazilian rice cultivar from 0.02 to 0.38 mg kg^−1^, while the grain Se contents increased in a smaller proportion, from 0.03 to 0.32 mg kg^−1^. According to the authors, the ingestion of the produced Se-biofortified rice would result in a daily Se intake of less than 16 µg day^−1^ [34], which is not enough to provide the recommended dietary intake of Se (55 µg day^−1^) [13]. Comparing the leaf Se contents reported by Reis et al. [34] with the results obtained in the present study, it can be assumed that rice grains from the control group would not supply daily Se requirements. However, leaf Se concentrations in the SeNP and Se(IV)-treated groups (at 0.5 mg L^−1^) were 2.2- and 2.6-fold higher than those reported by Reis et al. [34] for the Se(VI)-treated group. Thus, these treatments have the potential to produce Se-biofortified rice grains. Finally, at 5 mg L^−1^, SeNP and Se(IV) application resulted in 23- and 20-fold higher leaf Se content compared to Se(VI)-treated plants [34], which could produce grains that will exceed the tolerable Se intake limit (400 µg day^−1^) [13]. Of course, these are assumptions that will depend on the translocation rate of different Se forms to the grains, so further studies are needed to assess the feasibility of these treatments to produce Se-biofortified rice grains.

Interestingly, different patterns were observed for different Se formulations. Leaves exposed to SeNPs at 5 mg L^−1^ accumulated slightly more Se than those exposed to Se(IV). In the rice roots, an opposite trend was observed, i.e., a higher increase in Se concentrations when Se(IV) was applied compared to the application of SeNPs at the corresponding concentration (Figure 3B). In addition, for all treatments except the control group, the Se contents in leaves were higher than in roots.

These results may indicate a tendency for Se to accumulate in the aerial part when applied as SeNPs via foliar spray and a higher translocation to the roots whenis applied as Se(IV). A similar behavior was observed in the tomato plants, where the foliar application of SeNPs led to a higher Se accumulation in the fruits compared to the corresponding concentration of Se(VI) [33]. These authors concluded that SeNPs are more efficient for biofortification programs, while Se(VI) demonstrated potential toxicity at high doses [33]. In brown rice, the foliar application of SeNPs in plants grown in Cd- and Pb-contaminated soil caused significant grain Se enrichment [22]. However, in rice seedlings hydroponically exposed to SeNPs or Se salts (Se(IV) and Se(VI)), Wang et al. [35] observed that the Se was primarily accumulated in roots rather than in shoots, except for Se(VI) treatment. Therefore, foliar application seems to be more efficient than root exposure for grain biofortification.

#### 3.4.2. Ionomic Profile: Macro and Micronutrient Uptake and Accumulation

The application of SeNPs or Se(IV) in different concentrations induced significant changes in the concentrations of some nutrients in plant tissues, as can be seen in Table 2, which shows the concentrations of the macro- and micronutrients in the leaves and roots of rice seedlings after each treatment.

There was a slight increase in the average Na concentration in the roots and leaves after the application of SeNPs (at 0.5 or 5 mg L^−1^); however, this was not statistically significant. A possible reason was the use of Se(IV) in the SeNP synthesis, as Na could remain in the solution and be absorbed by the plant leaves. However, this increasing trend was not observed in the groups where Se(IV) was applied as a salt. Neither the Se source (SeNPs or Se(IV)), nor the applied concentration, affected the concentrations of Mg, Cu, Ca, K, and Zn in the rice roots and Mg, Mn, Cu, and Zn in the leaves.

The application of SeNPs at 5 mg L^−1^ and both Se(IV) treatments decreased the K concentration in the leaves compared to the group treated with SeNPs at 0.5 mg L^−1^, but no treatment was statistically different from the control group. A similar trend was observed for Co as its concentration in the roots and leaves decreased following the application of SeNPs at 5 mg L^−1^ or Se(IV) at 0.5 and 5 mg L^−1^, with the lowest Co content observed when Se(IV) was applied at 5 mg L^−1^. Interestingly, the same treatments that decreased K and Co concentration significantly increased the Ca content in the leaves. The application of Se(IV) at 0.5 mg L^−1^ decreased the Mn concentration in the roots compared to the control group.

The observed changes in the elemental concentrations (Table 2) could be related to the changes in the plant biomass since Se application reduced the root and shoot dry weight, as discussed in Section 3.2. Table S3 shows the total element accumulation in the roots and shoots per pot. No significant changes were observed between the control and treatment groups for the T_Ca_ in the leaves despite the observed increased Ca concentration in the leaves with Se addition. This can be explained by the loss of biomass. In other words, the total amount of Ca was similar per plant, but the smaller plants had a higher concentration when reported as a concentration. Conversely, for the Mn in the roots, the T_Mn_ was significantly lower for all Se-treated groups. A similar trend was observed for the T_Co_ in the roots and leaves, thus confirming that Se application reduces Mn and Co uptake by the rice roots. On the other hand, some of the elements whose concentrations did not change with Se treatments presented significant changes in total element content (Table S3). This applied in the case of Na (in leaves), as well as for the Mg, Cu, and Zn in the roots and leaves. The total content of these elements decreased following Se treatments, but this was masked by the loss of biomass.

Both antagonistic or synergistic effects with other elements have been reported in plants exposed to Se, which can be influenced by Se concentration, source, and application form [19,36,37]. According to Boldrin et al. [19], the foliar application of Se as Se(IV) or Se(VI) did not influence the Mg, Zn, and Mn content in the rice grains. On the other hand, the authors observed a reduction in the Cu content in the grains following foliar Se(IV) application [19]. The combined application of Se(VI) and N in the rice plants led to increased concentrations of N, P, K, Ca, Se, B, Cu, Zn, and Mo in the grains without affecting the concentrations of Mg and Mn [34]. In the leaves, increased concentrations of N, K, Cu, and Se, as well as decreased concentration of Ca, were reported in the same study [34]. The application of SeNPs or Se(VI) at 3 mg L^−1^ in tomato plants increased the Mg, Fe, and Zn concentration in fruits, while higher Se concentration (10 mg L^−1^) decreased the concentration of those nutrients [33].

The application of SeNPs at 0.5 mg L^−1^ was the only treatment that did not cause significant variations in the concentration of any macro or micronutrients in the roots and leaves compared to the control group. Therefore, it may be a suitable concentration for the agronomic biofortification of rice with Se, without affecting the content of other elements in the plant. As discussed in Section 3.4.1, an increase of 420% in leaf Se concentration was obtained with SeNP application at 0.5 mg L^−1^. Future studies with rice cultivation until grain maturity are needed to evaluate the potential of this treatment to produce Se-fortified grains in addition to evaluating how this treatment may affect the grain nutrient concentrations.

#### 3.4.3. As, Cd, and Pb Uptake and Accumulation

**Figure 4:**
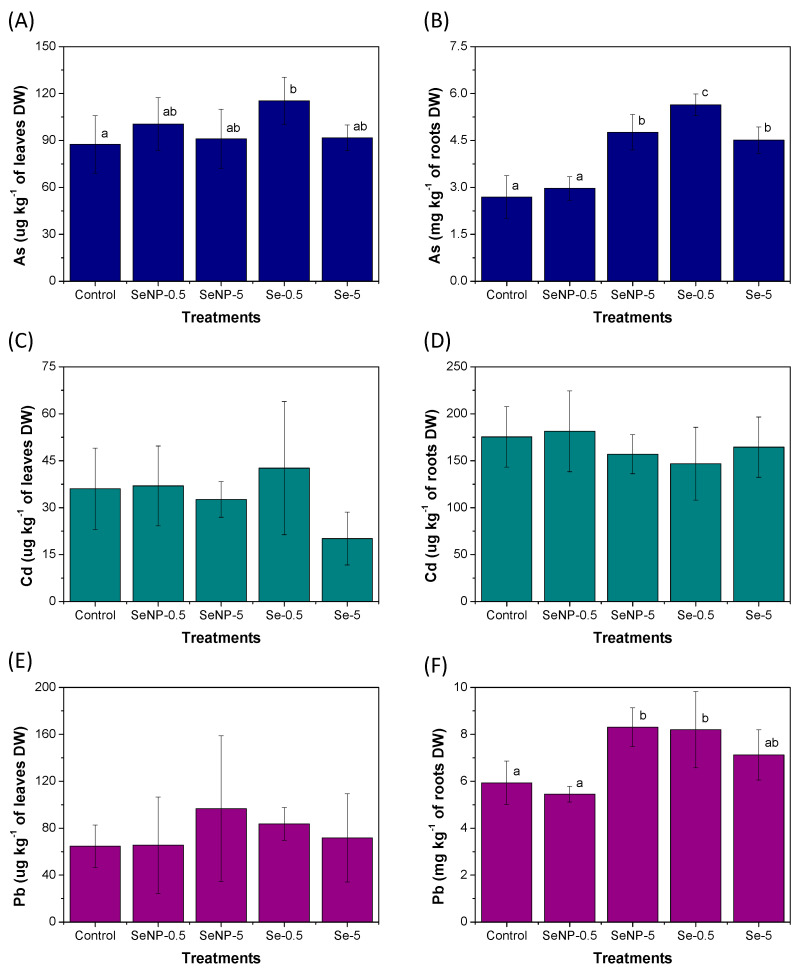
Concentrations of As (**A**,**B**), Cd (**C**,**D**), and Pb (**E**,**F**) in the leaves (**A**,**C**,**E**) and roots (**B**,**D**,**F**) of rice seedlings after 45 days of cultivation under foliar applications of the treatments. The data presented are the means of six replicates, while the error bar represents the standard deviation. Different letters represent a significant difference between the treatments (*p* < 0.05) for each variable. The treatment groups were as follows: control; SeNP−0.5: application of selenium nanoparticles at a concentration of 0.5 mg L^−1^ of Se; SeNP−5: application of selenium nanoparticles at a concentration of 5.0 mg L^−1^ of Se; Se−0.5: application of sodium selenite at a concentration of 0.5 mg L^−1^ of Se; and Se−5: application of sodium selenite at a concentration of 5.0 mg L^−1^ of Se.

Figure 4 shows the concentrations of the PTEs in plant tissues after each treatment. None of the treatments significantly impacted the Cd concentration in the leaves and roots (Figure 4C,D) or the Pb concentrations in the leaves (Figure 4E). Nonetheless, the application of Se(IV) at 5 mg L^−1^ slightly decreased the Cd concentration in the leaves from 36.0 ± 13.0 µg kg^−1^ in the control group to 20.1 ± 8.4 µg kg^−1^.

Regarding the As in the leaves and roots (Figure 4A,B) and the Pb in the roots (Figure 4F), significant differences were observed between the treatments. Compared to the control group, the As concentration in the leaves significantly increased following plant exposure to Se(IV) at 0.5 mg L^−1^. In the roots, the groups treated with SeNPs at 5 mg L^−1^ and both Se(IV) concentrations showed higher As concentrations than the control group and the group exposed to 0.5 mg L^−1^ of SeNPs. The highest As concentration in the roots was observed after the application of 0.5 mg L^−1^ of Se(IV) (5.6 ± 0.3 mg kg^−1^), which was twice the concentration of the control group (2.7 ± 0.7 mg kg^−1^), thus suggesting that Se(IV) stimulated the As uptake through the roots. This was confirmed by T_As_ calculation (Table S3), as the T_As_ in the roots increased following exposure to 0.5 mg L^−1^ of Se(IV). Considering that, in this experiment, the plants grew in soil that was not contaminated with As, as shown in Table 1, the absorbed As must have been naturally present in the soil. The other treatments did not significantly influence the T_As_ accumulation in the roots.

Previous studies have reported that ionic Se stimulates As uptake but seems to reduce its translocation from roots to shoots. Camara et al. [20] evaluated the effects of Se on the uptake and translocation of As in rice seedlings. The authors observed that the As content increased in rice roots but decreased in shoots with co-exposure to Se(IV) and arsenite. Similarly, Wang et al. [21] reported that Se(IV) stimulated As uptake by the rice roots but reduced shoot As content, while SeNPs (30 µmol L^−1^, or 2.37 mg L^−1^) did not affect As uptake. Paniz et al. [38] observed that Se biofortification with Se(VI) reduced As accumulation in rice grains, thus mitigating the risk of As toxicity in rice for human consumption. A possible reason for the observed results is a synergic effect between arsenite and Se(IV) uptake, but the exact mechanism of this process is still unclear [20,21,39].

Similar to As, the Pb concentrations in the rice roots increased in the groups treated with SeNPs at 5 mg L^−1^ and Se(IV) at 0.5 mg L^−1^, but the T_Pb_ did not change in these groups compared to the control. Wang et al. [22] reported that increasing the concentration of SeNPs (50–100 µmol L^−1^, or 3.95–7.90 mg L^−1^) promoted Pb accumulation in leaves. A similar behavior was observed in the roots in the present study, as the application of 5 mg L^−1^ of SeNPs caused an increase in the Pb concentration compared to the application of 0.5 mg L^−1^. However, the results of the T_Pb_ content indicate that the observed effect is related to the loss of biomass. The T_Pb_ accumulation in the roots decreased in the 0.5 mg L^−1^ SeNP-treated plants (Table S3), thus suggesting a protective effect of SeNPs at low concentrations.

Therefore, the results evidenced that the foliar application of Se(IV) or SeNPs induced changes in the uptake and accumulation of PTEs in the plant tissues. Interestingly, by increasing the applied concentration of Se(IV) from 0.5 to 5 mg L^−1^, the concentrations of As and Pb, as well as T_As_ and T_Pb_, tended to decline in the roots and leaves, thus reinforcing the effect of the concentration on the antagonistic or synergistic interaction between elements. In fact, a dual effect of Se on As uptake has been reported elsewhere, with an evident antagonism between As and Se at high concentrations (5 mg L^−1^) [40]. This antagonism may occur because arsenite and Se(IV) share the same Si transporter [20,21]. The arsenate is absorbed by phosphate transporters, which also partially mediate Se(IV) uptake [41,42], then competition can occur during plant uptakes of As and Se.

The same treatments that increased As and Pb concentrations in the roots decreased T_Cd_ accumulation. In the present study, although no significant differences were observed in the Cd concentrations in the roots, most likely due to biomass reduction, the application of Se(IV) or SeNPs (at 5 mg L^−1^) decreased the T_Cd_ accumulation in the roots (Table S3), which could protect rice plants from Cd oxidative stress. Similar to the results observed in this study, the literature shows that the application of Se (IV) or SeNPs reduces Cd uptake and accumulation in rice plants [22,43]. Foliar applications of 25 µmol L^−1^ of SeNPs (1.97 mg L^−1^) in rice plants grown in Cd- and Pb-contaminated soil slightly enhanced the Cd content in the leaves, while higher SeNP concentrations (above 50 µmol L^−1^, or 3.95 mg L^−1^) decreased Cd accumulation [22]. The mechanisms responsible for this are still unclear [22].

The application of SeNPs at 0.5 mg L^−1^ did not significantly influence (*p* > 0.05) the concentrations of the As, Cd, or Pb in any of the rice tissues, but it did decrease the T_Pb_ accumulation in the roots. Considering that PTEs can inhibit plant growth and development, as well as even harm photosynthesis [20,44,45] in addition to accumulating in edible plant parts and representing a potential risk to human health, the application of SeNPs at 0.5 mg L^−1^ seems to be safe from a food safety perspective.

### 3.5. Effect of Se on the Element Translocation from Roots to Shoots

The TF is defined as the ratio of the elemental content in the shoot to the root, and it is used to evaluate the translocation potential of the PTE upward [20,46]. The results of the As, Cd, Pb, and Se TFs in the rice seedlings under different Se treatments are summarized in Table 3. The TF presented in the following order: Se > Cd > As > Pb. This result is in accordance with a previous report that determined the soil-shoot TF for Cd, As, and Pb in rice plants [47].

No significant changes in either Cd or Pb TF, owing to these treatments, were observed. However, a slight increase in Cd TF was noticed following Se(IV) application at 0.5 mg L^−1^, which then decreased for the highest Se(IV) concentration. Under Cd stress, the exposure of rice plants to Se(IV) lowered the Cd TF by 66% relative to the treatment with Cd alone [46]. For Pb, the calculated TFs were extremely low, which indicates that this element accumulates in rice roots and is seldom transported to the aerial parts.

The As TF significantly decreased for Se(IV) treatments, as well as for SeNP application at 5 mg L^−1^. The As TF was similar between these three treatments (0.019–0.020); however, they were approximately 1.75-fold lower than those resulting from the control plants or SeNP treatment at 0.5 mg L^−1^ (0.034–0.036). The decrease in As TF for the aforementioned groups helps to explain the more pronounced increase observed in the As concentrations in the roots than in the leaves, as discussed in Section 3.4.3. These low TFs indicate that, when rice plants are exposed to Se(IV) or high concentrations of SeNPs, the absorbed As is primarily distributed in the roots rather than in the shoots. Previous studies have shown that Se(IV) and SeNPs reduce root-to-shoot As translocation in rice plants [20,21,48]. At a lower dose, the stronger inhibiting effect of Se(IV) on As translocation compared to SeNPs was observed in this study, which is in agreement with the results reported by Wang et al. [21]. However, the results observed herein do not agree with Camara et al. [20] in the fact that As translocation decreases with increasing Se(IV) dosage.

At the lower doses of Se(IV) or SeNPs, the root-to-shoot Se TF was 1.64–1.97, whereas it was only 0.41 in the control group. Meanwhile, when higher Se concentrations were supplied to the plants, Se TF increased to 4.47 (Se(IV)) or 6.61 (SeNPs), i.e., 11- and 16-fold increases, respectively, compared to the control group. These results confirm that the foliar application of Se, whether in ionic or nanoparticulate form, increases in a dose-dependent manner the Se accumulation in the aerial part, which is reflected in higher TF. As the Se content in roots also increased following Se(IV) or SeNP exposure (Figure 3B), the Se seems to be translocated from the shoots to the roots when applied as SeNPs (at 5 mg L^−1^) or Se(IV). A recent study demonstrated that Se is transported downward in plants when SeNPs are sprayed onto the leaves [11].

Furthermore, the application of SeNPs caused a higher increase in the Se TF in relation to the same Se concentrations as Se(IV). These results agree with those discussed in Section 3.4.1, thus confirming that Se tends to remain in the aerial part when applied as SeNPs, and its translocation rate to the roots was lower compared to Se(IV). Meanwhile, when applied as Se(IV), the Se translocation to the roots was more significant. It is already known that Se translocation from roots to shoots is influenced by its formulation [49]. The results of the present study indicate that the Se translocation downward is also influenced by the supplied form.

## 4. Conclusions

This study demonstrated that foliar applications of SeNPs at low doses (0.5 mg L^−1^) in rice seedlings increased by 420% the leaf Se concentration and reduces the total Pb accumulation in roots. Moreover, this treatment did not significantly affect the concentration of photosynthetic pigments, nutrients, and PTEs, or the plant’s agronomic parameters, except for a reduction in root dry weight. Meanwhile, the foliar application of SeNPs at 5 mg L^−1^, or Se(IV), inhibited plant growth and increased the concentrations of As and Pb in the rice roots, but it also decreased T_Cd_ accumulation in the roots. At 0.5 mg L^−1^, Se(IV) significantly increased root As uptake. Results suggest a higher phytotoxic effect of Se(IV) compared to SeNPs on rice seedlings. A comparison of the ionic and nanoparticulate formulations indicated a tendency for the Se to remain in the aerial part when applied as SeNPs and as a higher translocation to the roots when applied as Se(IV). Based on these results, the foliar application of the prepared SeNPs at 0.5 mg L^−1^ may be more effective for the biofortification of rice grains than the conventional ionic formulation since Se could remain in the aerial part and reach the grains without affecting the development of plants under evaluated conditions. The present study contributes to the knowledge about the use of NPs as fertilizers, thus providing additional evidence that nanobiotechnology is a suitable tool for improving agriculture. Future studies are needed to understand if Se remains in the nanoform or is converted into dissolved selenides. In addition, studies with rice plants cultivated until grain maturation are required to evaluate whether, at this optimal concentration of SeNPs, it is possible to observe grain Se enrichment.

## Figures and Tables

**Table 1:** Summary of the soil properties.

Total sand (g kg^−1^)	Coarse sand (g kg^−1^)	Fine sand (g kg^−1^)	Clay (g kg^−1^)	Silt (g kg^−1^)	pH-CaCl_2_
256	136	120	583	161	5.9
OM (g kg^−1^)	SB (mmolc dm^−3^)	K-Exc (mmolc dm^−3^)	Ca- Exc (mmolc dm^−3^)	Mg- Exc (mmolc dm^−3^)	H + Al (mmolc dm^−3^)
40	116.6	7.6	94	15	24
CEC (mmolc dm^−3^)	V (%)	EC (ds m^−1^)	C (g dm^−3^)	P (resin) (mg kg^−1^)	Co (µg kg^−1^)
141	83	1.66	23	57	657 ± 122
Cu (mg kg^−1^)	Zn (mg kg^−1^)	As (mg kg^−1^)	Se (mg kg^−1^)	Cd (µg kg^−1^)	Pb (mg kg^−1^)
10.4 ± 1.4	23.4 ± 2.1	4.11 ± 0.34	6.49 ± 0.48	27.5 ± 4.5	9.98 ± 1.60

OM: organic matter; SB: sum of bases equal to exchangeable Ca, Mg, and K; Exc: exchangeable; CEC: cation exchange capacity (millimol of charge per dm^3^) and equal to SB + H + Al; V: base saturation and equal to (100 × SB)/CEC; and EC: electrical conductivity.

**Table 2:** Concentrations of the macro and micronutrients in the leaves and roots of the rice seedlings after 45 days of cultivation under foliar applications of the treatments. The data are presented as the mean ± standard deviation (n = 6). Different superscript letters represent a significant difference between the treatments (*p* < 0.05) for each variable. The treatment groups were as follows: control; SeNP−0.5: application of selenium nanoparticles at a concentration of 0.5 mg L^−1^ of Se; SeNP−5: application of selenium nanoparticles at a concentration of 5.0 mg L^−1^ of Se; Se−0.5: application of sodium selenite at a concentration of 0.5 mg L^−1^ of Se; and Se−5: application of sodium selenite at a concentration of 5.0 mg L^−1^ of Se.

Element (conc.)	Tissue	Control	SeNP-0.5	SeNP-5	Se-0.5	Se-5
Na (mg kg^−1^)	Leaf	83 ± 28	117 ± 39	141 ± 147	99 ± 35	80 ± 24
Na (g kg^−1^)	Root	1.56 ± 0.27	1.82 ± 0.25	1.64 ± 0.25	1.47 ± 0.19	1.59 ± 0.34
Mg (g kg^−1^)	Leaf	1.85 ± 0.19	1.75 ± 0.14	1.63 ± 0.20	1.58 ± 0.22	1.65 ± 0.24
Mg (g kg^−1^)	Root	1.57 ± 0.32	1.50 ± 0.22	1.30 ± 0.17	1.47 ± 0.19	1.49 ± 0.13
K (g kg^−1^)	Leaf	31.9 ± 1.3 ^ab^	35.9 ± 3.0 ^a^	30.6 ± 2.0 ^b^	29.6 ± 3.1 ^b^	29.7 ± 2.3 ^b^
K (g kg^−1^)	Root	29.7 ± 3.4	33.8 ± 3.1	32.1 ± 3.3	31.0 ± 4.1	32.8 ± 3.7
Ca (g kg^−1^)	Leaf	3.72 ± 0.45 ^a^	3.22 ± 0.40 ^a^	4.93 ± 0.89 ^b^	5.10 ± 0.41 ^b^	4.93 ± 0.47 ^b^
Ca (g kg^−1^)	Root	2.55 ± 0.33	2.15 ± 0.20	2.51 ± 0.11	2.65 ± 0.37	2.49 ± 0.46
Mn (mg kg^−1^)	Leaf	755 ± 113	659 ± 144	633 ± 95	621 ± 111	648 ± 128
Mn (mg kg^−1^)	Root	181 ± 49 ^a^	167 ± 29 ^ab^	158 ± 12 ^ab^	131 ± 9 ^b^	147 ± 21 ^ab^
Co (µg kg^−1^)	Leaf	19.9 ± 4.9 ^a^	17.4 ± 3.3 ^ab^	13.5 ± 3.0 ^b^	13.0 ± 2.5 ^b^	12.3 ± 2.1 ^b^
Co (µg kg^−1^)	Root	736 ± 114 ^a^	628 ± 52 ^ab^	490 ± 40 ^b^	515 ± 103 ^b^	463 ± 89 ^bc^
Cu (mg kg^−1^)	Leaf	7.34 ± 0.57	7.63 ± 0.52	6.92 ± 0.76	6.83 ± 0.67	7.18 ± 0.40
Cu (mg kg^−1^)	Root	18.9 ± 3.5	14.0 ± 1.4	16.1 ± 3.3	16.5 ± 7.6	15.8 ± 4.1
Zn (mg kg^−1^)	Leaf	34.3 ± 3.0	32.3 ± 3.5	29.7 ± 3.8	31.4 ± 3.7	30.3 ± 2.2
Zn (mg kg^−1^)	Root	56.5 ± 6.1	54.3 ± 6.6	59.3 ± 8.0	63.4 ± 9.8	56.2 ± 8.8

**Table 3:** Effects of the Se treatments on the transfer factor of Se, As, Cd, and Pb in the rice seedlings. The data are presented as the mean ± standard deviation (n = 6). Different superscript letters represent a significant difference between the treatments (*p* < 0.05) for each variable. The treatment groups were as follows: control; SeNP−0.5: application of selenium nanoparticles at a concentration of 0.5 mg L^−1^ of Se; SeNP−5: application of selenium nanoparticles at a concentration of 5.0 mg L^−1^ of Se; Se−0.5: application of sodium selenite at a concentration of 0.5 mg L^−1^ of Se; and Se−5: application of sodium selenite at a concentration of 5.0 mg L^−1^ of Se.

Element	Control	SeNP-0.5	SeNP-5	Se-0.5	Se-5
As	0.036 ± 0.016 ^a^	0.034 ± 0.002 ^a^	0.019 ± 0.004 ^b^	0.020 ± 0.003 ^b^	0.020 ± 0.003 ^b^
Cd	0.21 ± 0.09	0.20 ± 0.05	0.21 ± 0.04	0.34 ± 0.29	0.13 ± 0.05
Pb	0.011 ± 0.004	0.012 ± 0.007	0.012 ± 0.008	0.010 ± 0.002	0.010 ± 0.004
Se	0.41 ± 0.09 ^a^	1.97 ± 0.43 ^b^	6.61 ± 1.23 ^c^	1.64 ± 0.10 ^b^	4.47 ± 0.94 ^d^

## Data Availability

The raw data supporting the conclusions of this article will be made available by the authors on request.

## Supplementary Materials

The following supporting information can be downloaded at: https://www.mdpi.com/article/10.3390/toxics12070482/s1, Figure S1: Transmission electron microscopy image of SeNPs showing spherical and well-dispersed nanoparticles. The white bar indicates 500 nm; Figure S2: Size distributions of SeNPs diluted in ultrapure water (red line) or 0.1% m/v Triton X-100 (green line) obtained by dynamic light scattering; Figure S3: Representative images of rice seedlings after 45 days of cultivation. Groups: (A) control; (B) SeNPs at 0.5 mg L^−1^; (C) SeNPs at 5.0 mg L^−1^; (D) sodium selenite at 0.5 mg L^−1^; and (E) sodium selenite at 5.0 mg L^−1^; Table S1: ICP-MS operating conditions for the total element determination in rice tissues; Table S2: Concentrations of analytes (µg kg^−1^) and recovery percentages obtained for standard reference materials of water (NIST 1640a), plants (BCR-670, C1003a, and NIST 1573a), and soil (CRM049), as analyzed by ICP-MS. The results are expressed as mean ± standard deviation, or as the confidence interval for the indicative values (non-certified) of the analyte concentrations; Table S3: The total element accumulation in the leaves and roots of the rice seedlings after 45 days of cultivation under foliar applications of the treatments. The data are presented as the mean ± standard deviation (n = 6). Different superscript letters represent a significant difference between treatments (*p* < 0.05) for each variable. The treatment groups were as follows: control; SeNP-0.5: application of selenium nanoparticles at a concentration of 0.5 mg L^−1^ of Se; SeNP-5: application of selenium nanoparticles at a concentration of 5.0 mg L^−1^ of Se; Se-0.5: application of sodium selenite at a concentration of 0.5 mg L^−1^ of Se; and Se-5: application of sodium selenite at a concentration of 5.0 mg L^−1^ of Se.

## References

[1] Lal R. (2015). Restoring soil quality to mitigate soil degradation. Sustainability.

[2] Rehmanullah, Muhammad Z., Inayat N., Majeed A., Rakshit A., Singh H., Singh A., Singh U., Fraceto L. (2020). Application of nanoparticles in agriculture as fertilizers and pesticides: Challenges and opportunities. New Frontiers in Stress Management for Durable Agriculture.

[3] FAO, IFAD, UNICEF, WFP, WHO (2019). The State of Food Security and Nutrition in the World 2019. Safeguarding against Economic Slowdowns and Downturns.

[4] Freitas D.C., de Andrade A.M., da Costa L.F., Azevedo R.A., Arruda M.A. (2021). There is plenty of room at the plant science: A review of nanoparticles applied to plant cultures. Ann. Appl. Biol..

[5] Paramo L.A., Feregrino-Pérez A.A., Guevara R., Mendoza S., Esquivel K. (2020). Nanoparticles in agroindustry: Applications, toxicity, challenges, and trends. Nanomaterials.

[6] Freire B.M., Lange C.N., Cavalcanti Y.T., Monteiro L.R., Pieretti J.C., Seabra A.B., Batista B.L. (2024). The dual effect of Selenium nanoparticles in rice seedlings: From increasing antioxidant activity to inducing oxidative stress. Plant Stress.

[7] Kohatsu M.Y., Pelegrino M.T., Monteiro L.R., Freire B.M., Pereira R.M., Fincheira P., Rubilar O., Tortella G., Batista B.L., de Jesus T.A. (2021). Comparison of foliar spray and soil irrigation of biogenic CuO nanoparticles (NPs) on elemental uptake and accumulation in lettuce. Environ. Sci. Pollut. Res..

[8] Pelegrino M.T., Pieretti J.C., Lange C.N., Kohatsu M.Y., Freire B.M., Batista B.L., Fincheira P., Tortella G.R., Rubilar O., Seabra A.B. (2021). Foliar spray application of CuO nanoparticles (NPs) and S-nitrosoglutathione enhances productivity, physiological and biochemical parameters of lettuce plants. J. Chem. Technol. Biotechnol..

[9] Rizwan M., Ali S., ur Rehman M.Z., Malik S., Adrees M., Qayyum M.F., Alamri S.A., Alyemeni M.N., Ahmad P. (2019). Effect of foliar applications of silicon and titanium dioxide nanoparticles on growth, oxidative stress, and cadmium accumulation by rice (*Oryza sativa*). Acta Physiol. Plant..

[10] Moreno-Martín G., Sanz-Landaluze J., León-González M.E., Madrid Y. (2020). Insights into the accumulation and transformation of Ch-SeNPs by *Raphanus sativus* and *Brassica juncea*: Effect on essential elements uptake. Sci. Total Environ..

[11] Wang Y., Feng L.J., Sun X.D., Zhang M., Duan J.L., Xiao F., Lin W., Zhu F.P., Kong X.P., Ding Z. (2023). Incorporation of selenium derived from nanoparticles into plant proteins in vivo. ACS Nano.

[12] Rayman M.P. (2012). Selenium and human health. Lancet.

[13] Institute of Medicine (IOM), The National Academies (2000). Dietary Reference Intakes for Vitamin C, Vitamin E, Selenium and Carotenoids.

[14] Jones G.D., Droz B., Greve P., Gottschalk P., Poffet D., McGrath S.P., Seneviratne S.I., Smith P., Winkel L.H.E. (2017). Selenium deficiency risk predicted to increase under future climate change. Proc. Natl. Acad. Sci. USA.

[15] Galić L., Vinković T., Ravnjak B., Lončarić Z. (2021). Agronomic biofortification of significant cereal crops with selenium—A review. Agronomy.

[16] El-Ramady H.R., Domokos-Szabolcsy É., Abdalla N.A., Alshaal T.A., Shalaby T.A., Sztrik A., Prokisch J., Fári M. (2014). Selenium and nano-selenium in agroecosystems. Environ. Chem. Lett..

[17] Wang M., Zhou F., Cheng N., Chen P., Ma Y., Zhai H., Qi M., Liu N., Liu Y., Meng L. (2022). Soil and foliar selenium application: Impact on accumulation, speciation, and bioaccessibility of selenium in wheat (*Triticum aestivum* L.). Front. Plant Sci..

[18] de Brito Mateus M.P., Tavanti R.F.R., Tavanti T.R., Santos E.F., Jalal A., Dos Reis A.R. (2021). Selenium biofortification enhances ROS scavenge system increasing yield of coffee plants. Ecotoxicol. Environ. Saf..

[19] Boldrin P.F., Faquin V., Ramos S.J., Boldrin K.V.F., Ávila F.W., Guilherme L.R.G. (2013). Soil and foliar application of selenium in rice biofortification. J. Food Compos. Anal..

[20] Camara A.Y., Wan Y., Yu Y., Wang Q., Li H. (2018). Effect of selenium on uptake and translocation of arsenic in rice seedlings (*Oryza sativa* L.). Ecotoxicol. Environ. Saf..

[21] Wang K., Wang Y., Wan Y., Mi Z., Wang Q., Wang Q., Li H. (2021). The fate of arsenic in rice plants (*Oryza sativa* L.): Influence of different forms of selenium. Chemosphere.

[22] Wang C., Cheng T., Liu H., Zhou F., Zhang J., Zhang M., Liu X., Shi W., Cao T. (2021). Nano-selenium controlled cadmium accumulation and improved photosynthesis in indica rice cultivated in lead and cadmium combined paddy soils. J. Environ. Sci..

[23] Freire B.M., Cavalcanti Y.T., Lange C.N., Pieretti J.C., Pereira R.M., Gonçalves M.C., Nakazato G., Seabra A.B., Batista B.L. (2022). Evaluation of collision/reaction gases in single-particle ICP-MS for sizing selenium nanoparticles and assessment of their antibacterial activity. Nanotechnology.

[24] da Silva F.C. (2009). Manual de análises químicas de solos, plantas e fertilizantes. Embrapa Informação Tecnológica.

[25] Raij B.v., Andrade J.C., Cantarella H., Quaggio J.A. (2001). Análise Química para Avaliação da Fertilidade de Solos Tropicais.

[26] Boonyanitipong P., Kositsup B., Kumar P., Baruah S., Dutta J. (2011). Toxicity of ZnO and TiO_2_ nanoparticles on germinating rice seed *Oryza sativa* L.. Int. J. Biosci. Biochem. Bioinform..

[27] Zahedi S.M., Abdelrahman M., Hosseini M.S., Hoveizeh N.F., Tran L.S.P. (2019). Alleviation of the effect of salinity on growth and yield of strawberry by foliar spray of selenium-nanoparticles. Environ. Pollut..

[28] Lichtenthaler H.K., Wellburn A.R. (1983). Determinations of total carotenoids and chlorophylls a and b of leaf extracts in different solvents. Biochem. Soc. Trans..

[29] Paniz F.P., Pedron T., Freire B.M., Torres D.P., Silva F.F., Batista B.L. (2018). Effective procedures for the determination of As, Cd, Cu, Fe, Hg, Mg, Mn, Ni, Pb, Se, Th, Zn, U and rare earth elements in plants and foodstuffs. Anal. Methods.

[30] (2016). Guidance on Validation of Analytical Methods, Revision 5 August 2016.

[31] Fidelis R.R., Campestrini R., Martinez R.A.S., de Oliveira Tavares T.C., Lopes M.B.S. (2018). Physiological quality of rice in the function of selenium doses. Rev. Agric. Neotrop..

[32] Hopper J.L., Parker D.R. (1999). Plant availability of selenite and selenate as influenced by the competing ions phosphate and sulfate. Plant Soil.

[33] Neysanian M., Iranbakhsh A., Ahmadvand R., Ardebili Z.O., Ebadi M. (2020). Comparative efficacy of selenate and selenium nanoparticles for improving growth, productivity, fruit quality, and postharvest longevity through modifying nutrition, metabolism, and gene expression in tomato; potential benefits and risk assessment. PLoS ONE.

[34] Reis H.P.G., de Queiroz Barcelos J.P., Junior E.F., Santos E.F., Silva V.M., Moraes M.F., Putti F.F., dos Reis A.R. (2018). Agronomic biofortification of upland rice with selenium and nitrogen and its relation to grain quality. J. Cereal Sci..

[35] Wang K., Wang Y., Li K., Wan Y., Wang Q., Zhuang Z., Guo Y., Li H. (2020). Uptake, translocation and biotransformation of selenium nanoparticles in rice seedlings (*Oryza sativa* L.). J. Nanobiotechnol..

[36] Pazurkiewicz-Kocot K., Kita A., Pietruszka M. (2008). Effect of selenium on magnesium, iron, manganese, copper, and zinc accumulation in corn treated by indole-3-acetic acid. Commun. Soil Sci. Plant Anal..

[37] Feng R., Wei C., Tu S., Wu F. (2009). Effects of Se on the uptake of essential elements in *Pteris vittata* L.. Plant Soil.

[38] Paniz F.P., Pedron T., Procópio V.A., Lange C.N., Freire B.M., Batista B.L. (2023). Selenium Biofortification Enhanced Grain Yield and Alleviated the Risk of Arsenic and Cadmium Toxicity in Rice for Human Consumption. Toxics.

[39] Bluemlein K., Klimm E., Raab A., Feldmann J. (2009). Selenite enhances arsenate toxicity in *Thunbergia alata*. Environ. Chem..

[40] Han D., Xiong S., Tu S., Liu J., Chen C. (2015). Interactive effects of selenium and arsenic on growth, antioxidant system, arsenic and selenium species of *Nicotiana tabacum* L.. Environ. Exp. Bot..

[41] Meharg A.A., Macnair M.R. (1992). Suppression of the high affinity phosphate uptake system: A mechanism of arsenate tolerance in *Holcus lanatus* L.. J. Exp. Bot..

[42] Zhang L., Hu B., Li W., Che R., Deng K., Li H., Yu F., Ling H., Li Y., Chu C. (2014). OsPT2, a phosphate transporter, is involved in the active uptake of selenite in rice. New Phytol..

[43] Gao M., Zhou J., Liu H., Zhang W., Hu Y., Liang J., Zhou J. (2018). Foliar spraying with silicon and selenium reduces cadmium uptake and mitigates cadmium toxicity in rice. Sci. Total Environ..

[44] An M., Wang H., Fan H., Ippolito J.A., Meng C., Yulian E., Li Y., Wang K., Wei C. (2019). Effects of modifiers on the growth, photosynthesis, and antioxidant enzymes of cotton under cadmium toxicity. J. Plant Growth Regul..

[45] Cao F., Cai Y., Liu L., Zhang M., He X., Zhang G., Wu F. (2015). Differences in photosynthesis, yield and grain cadmium accumulation as affected by exogenous cadmium and glutathione in the two rice genotypes. Plant Growth Regul..

[46] Wan Y., Wang K., Liu Z., Yu Y., Wang Q., Li H. (2019). Effect of selenium on the subcellular distribution of cadmium and oxidative stress induced by cadmium in rice (*Oryza sativa* L.). Environ. Sci. Pollut. Res..

[47] Andrade G.F., Paniz F.P., Martins A.C., Rocha B.A., da Silva Lobato A.K., Rodrigues J.L., Cardoso-Gustavson P., Masuda H.P., Batista B.L. (2018). Agricultural use of Samarco’s spilled mud assessed by rice cultivation: A promising residue use?. Chemosphere.

[48] Camara A.Y., Wan Y., Yu Y., Wang Q., Wang K., Li H. (2019). Effect of endogenous selenium on arsenic uptake and antioxidative enzymes in as-exposed rice seedlings. Int. J. Environ. Res. Public Health.

[49] De Filippis L.F. (2010). Biochemical and molecular aspects in phytoremediation of selenium. Plant Adaptation and Phytoremediation.

