# Supplementary material for: Ionomic Profile of Rice Seedlings after Foliar Application of Selenium Nanoparticles
Source: Toxics. 2024 Jul 1;12(7):482. doi: 10.3390/toxics12070482 (PMC11281011; doi:10.3390/toxics12070482)
Supplement: Supplementary file 1 [file toxics-12-00482-s001.zip › toxics-3006911-supplementary.pdf]

Supplementary Information

Ionomic Profile of Rice Seedlings after Foliar Application of Selenium Nanoparticles

Bruna Moreira Freire [1,2,*], Camila Neves Lange [1], Yasmin Tavares Cavalcanti [1], Amedea Barozzi Seabra [1,3] and Bruno Lemos Batista [1,*]

[1] Center for Natural and Human Sciences (CCNH), Federal University of ABC (UFABC), Santo André 09210-580, São Paulo, Brazil; camila.lange@ufabc.edu.br (C.N.L.); yasmintata18@gmail.com (Y.T.C.); amedea.seabra@ufabc.edu.br (A.B.S.)

[2] Department of Analytical Chemistry, Aragon Institute of Engineering Research (I3A), University of Zaragoza, 50009 Zaragoza, Spain

[3] National Institute of Science and Technology in Nanotechnology for Sustainable Agriculture, INCTNanoAgro, Santo André 09210-580, São Paulo, Brazil

* Correspondence: bruna.freire@ufabc.edu.br (B.M.F.); bruno.lemos@ufabc.edu.br (B.L.B.)

Supplemental Figures

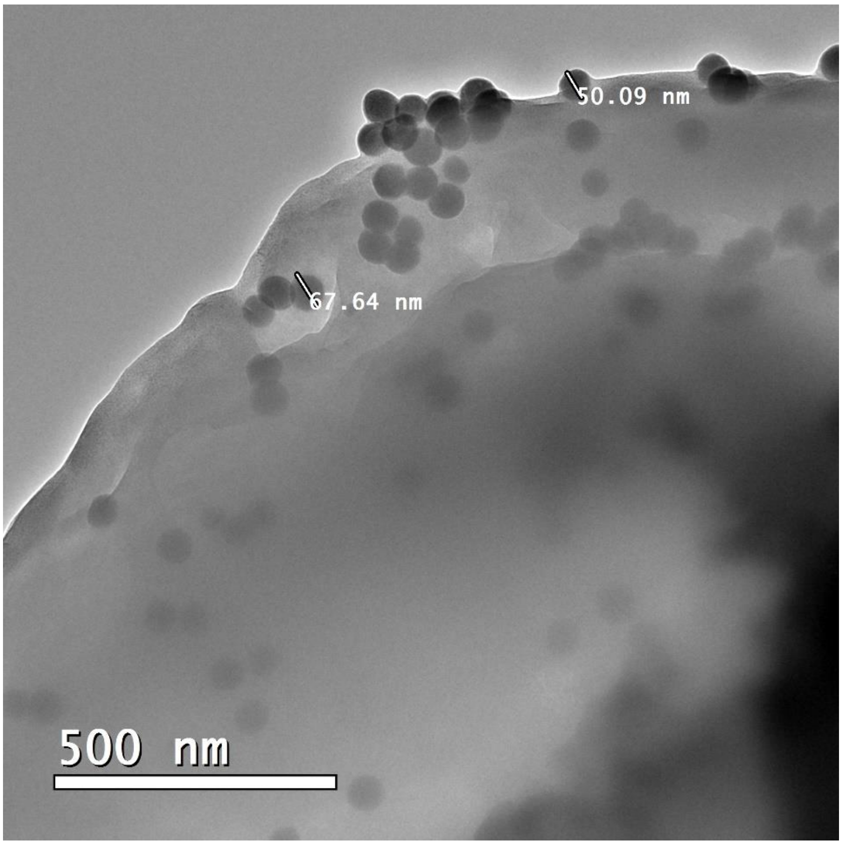

Figure S1. Transmission electron microscopy image of SeNPs showing spherical and well-dispersed nanoparticles. The white bar indicates 500 nm.

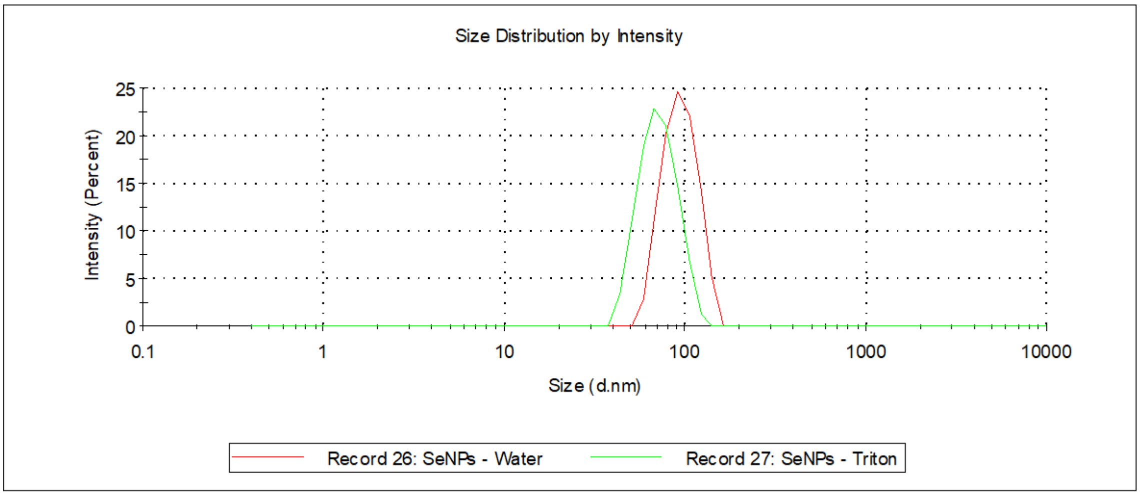

Figure S 2. Size distributions of SeNPs diluted in ultrapure water (red line) or 0.1 % m/v Triton X-100 (green line) obtained by dynamic light scattering.

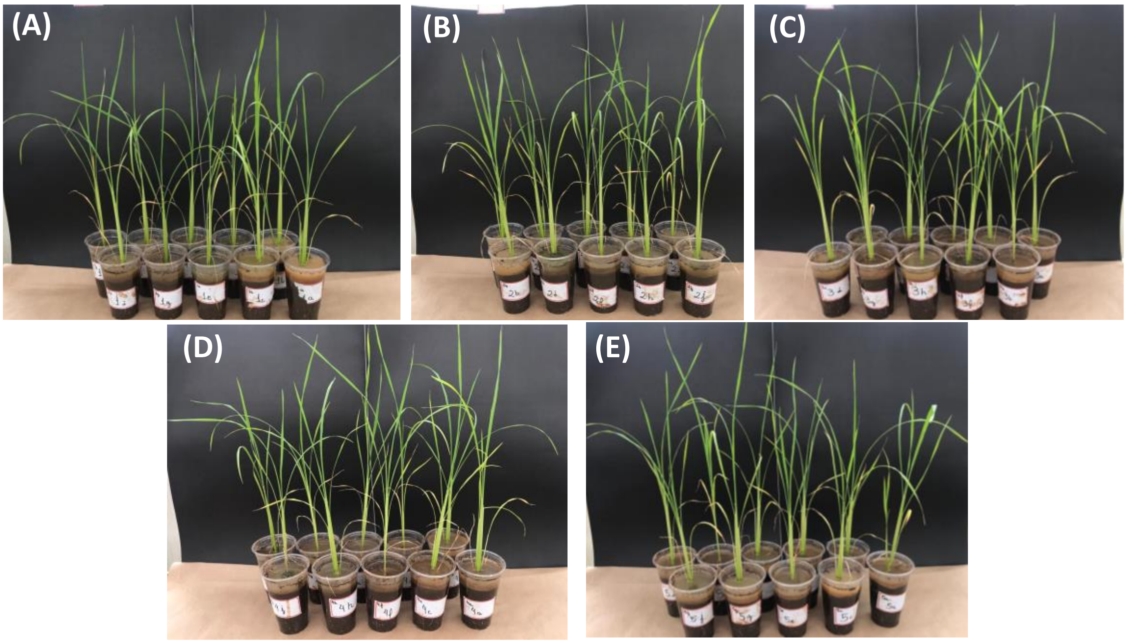

Figure S3. Representative images of rice seedlings after 45 days of cultivation. Groups: (**A**) Control; (**B**) SeNPs at 0.5 mg L^{-1}; (**C**) SeNPs at 5.0 mg L^{-1}; (**D**) Sodium Selenite at 0.5 mg L^{-1}; and (**E**) Sodium Selenite at 5.0 mg L^{-1}.

Supplemental Tables

Table S1. ICP-MS operating conditions for total element determination in rice tissues.

**Parameter**	**ICP-MS 7900**
Monitored isotopes and respective instrumental limit of detection (LoD, μg L^{-1})	^{23}Na (9.6), ^{24}Mg (0.31), ^{39}K (7.0), ^{44}Ca (4.0), ^{55}Mn (0.01), ^{59}Co (0.003), ^{65}Cu (0.16), ^{68}Zn (0.31), ^{75}As (0.03), ^{78}Se (0.04), ^{112}Cd (0.009), ^{208}Pb (0.05)
Internal standards (25 μg L^{-1})	^{74}Ge e ^{193}Ir
Nebulizer	Mira Mist™
Spray chamber	Scott (*double pass*)
RF power	1550 W
Nebulizer gas flow (Ar)	1.01 to 1.05 L min^{-1}
Plasma gas flow (Ar)	15.0 L min^{-1}
Elements measured in He mode	Mg, Co, Cu, Zn, As, Se, Cd, Pb
He flow in the collision cell	5 mL min^{-1}
Elements measured in HEHe mode	Na, K, Ca, Mn
He flow in the collision cell	10 mL min^{-1}
Elements measured in H_2 mode	Se
H_2 flow in the reaction cell	6 mL min^{-1}
Replicates	3

Table S2. Concentrations of analytes (μg kg^{-1}) and recovery percentages obtained for standard reference materials of water (NIST 1640a), plants (BCR-670, C1003a, and NIST 1573a), and soil (CRM049) analyzed by ICP-MS. Results are expressed as mean ± standard deviation, or as the confidence interval for the indicative values (non-certified) of analyte concentrations.

Element (unit)	Reference Material	Certified value	Found value	Recovery (%)
Se (μg L^{-1})	NIST 1640a	20.13 ± 0.17	18.68 ± 0.41	93
Se (μg kg^{-1})	BCR-670	149-273	157 ± 5	Within the range
Se (mg kg^{-1})	CRM049	203 ± 5.50	162 ± 11.8	80
Na (mg L^{-1})	NIST 1640a	3.137 ± 0.031	3.159 ± 0.012	101
Na (mg kg^{-1})	C1003a	2710 ± 840	2492 ± 112	92
Na (mg kg^{-1})	NIST 1573a	136 ± 4	159 ± 3	117
Mg (mg L^{-1})	NIST 1640a	1.0586 ± 0.0041	0.9821 ± 0.0151	93
Mg (mg kg^{-1})	C1003a	4110 ± 360	3701 ± 168	90
Mg (mg kg^{-1})	NIST 1573a	12000	9579 ± 355	80
K (μg L^{-1})	NIST 1640a	579.9 ± 2.3	559.9 ± 6.0	96
K (mg kg^{-1})	C1003a	43300 ± 3800	44880 ± 1947	104
K (mg kg^{-1})	NIST 1573a	27000 ± 500	25608 ± 320	95
Ca (mg L^{-1})	NIST 1640a	5.615 ± 0.021	6.689 ± 0.074	119
Ca (mg kg^{-1})	C1003a	27400 ± 4400	27312 ± 1022	100
Ca (mg kg^{-1})	NIST 1573a	50500 ± 900	47749 ± 753	94
Mn (μg L^{-1})	NIST 1640a	40.39 ± 0.36	39.48 ± 0.38	98
Mn (mg kg^{-1})	C1003a	470 ± 69	446 ± 17	95
Mn (mg kg^{-1})	NIST 1573a	246 ± 8	238 ± 5	97
Co (μg L^{-1})	NIST 1640a	20.24 ± 0.24	18.85 ± 0.31	93
Co (mg kg^{-1})	C1003a	0.33 ± 0.14	0.21 ± 0.007	64
Co (mg kg^{-1})	NIST 1573a	0.57 ± 0.02	0.50 ± 0.02	88
Co (mg kg^{-1})	CRM049	217 ± 4.77	195 ± 15	90
Cu (μg L^{-1})	NIST 1640a	85.75 ± 0.51	78.82 ± 1.27	92
Cu (mg kg^{-1})	C1003a	1130 ± 140	914 ± 33	81
Cu (mg kg^{-1})	NIST 1573a	4.70 ± 0.14	3.96 ± 0.12	84
Cu (mg kg^{-1})	BCR-670	1.82 ± 0.30	1.45 ± 0.04	80

Cu (mg kg^{-1})	CRM049	133 ± 2.52	113 ± 7.33	85
Zn (μg L^{-1})	NIST 1640a	55.64 ± 0.35	51.40 ± 1.84	92
Zn (mg kg^{-1})	C1003a	37.5 ± 5.0	32.2 ± 1.1	86
Zn (mg kg^{-1})	NIST 1573a	30.9 ± 0.7	27.4 ± 1.0	89
Zn (mg kg^{-1})	BCR-670	24.0 ± 2.1	20.6 ± 0.7	86
Zn (mg kg^{-1})	CRM049	433 ± 7.92	394 ± 25.9	91
As (μg L^{-1})	NIST 1640a	8.075 ± 0.070	7.446 ± 0.298	92
As (mg kg^{-1})	C1003a	19.0 ± 2.8	22.2 ± 0.9	117
As (μg kg^{-1})	NIST 1573a	112 ± 4	132 ± 9	118
As (mg kg^{-1})	BCR-670	1.98 ± 0.19	1.72 ± 0.07	87
As (mg kg^{-1})	CRM049	136 ± 2.49	108 ± 7.85	79
Cd (μg L^{-1})	NIST 1640a	3.992 ± 0.074	4.160 ± 0.080	104
Cd (mg kg^{-1})	C1003a	26.6 ± 2.1	24.1 ± 1.7	91
Cd (mg kg^{-1})	NIST 1573a	1.52 ± 0.04	1.48 ± 0.22	97
Cd (μg kg^{-1})	BCR-670	75.5 ± 2.5	87.5 ± 2.8	116
Cd (mg kg^{-1})	CRM049	87.9 ± 1.71	79.4 ± 4.57	90
Pb (μg L^{-1})	NIST 1640a	12.101 ± 0.050	11.373 ± 0.681	94
Pb (mg kg^{-1})	C1003a	3.67 ± 0.55	3.56 ± 0.20	97
Pb (mg kg^{-1})	BCR-670	2.06 ± 0.12	1.78 ± 0.19	86
Pb (mg kg^{-1})	CRM049	340 ± 7.24	282 ± 14.7	83

Table S3. Total element accumulation in leaves and roots of rice seedlings after 45 days of cultivation under foliar application of the treatments. The data is presented as the mean ± standard deviation (n=6). Different superscript letters represent a significant difference between treatments ($p < 0.05$) for each variable. The treatment groups are: Control; SeNP-0.5: application of selenium nanoparticles at a concentration of 0.5 mg L^{-1} of Se; SeNP-5: application of selenium nanoparticles at a concentration of 5.0 mg L^{-1} of Se; Se-0.5: application of sodium selenite at a concentration of 0.5 mg L^{-1} of Se; Se-5: application of sodium selenite at a concentration of 5.0 mg L^{-1} of Se.

Total Element (µg pot^{-1})	Tissue	Control	SeNP-0.5	SeNP-5	Se-0.5	Se-5
Se	Leaf	0.047 ± 0.010^{b}	0.216 ± 0.044^{b}	2.01 ± 0.22^{a}	0.226 ± 0.029^{b}	1.82 ± 0.25^{a}
Se	Root	0.20 ± 0.04^{c}	0.15 ± 0.03^{c}	0.42 ± 0.04^{b}	0.20 ± 0.06^{c}	0.59 ± 0.15^{a}
Na	Leaf	24 ± 7	30 ± 8	33 ± 35	22 ± 7	19 ± 6
Na	Root	759 ± 105^{a}	637 ± 92^{ab}	513 ± 69^{b}	482 ± 118^{b}	522 ± 99^{b}
Mg	Leaf	536 ± 69^{a}	455 ± 102^{ab}	370 ± 59^{b}	358 ± 63^{b}	394 ± 94^{b}
Mg	Root	769 ± 172^{a}	527 ± 111^{b}	413 ± 85^{b}	485 ± 129^{b}	497 ± 90^{b}
K	Leaf	9214 ± 259^{a}	9234 ± 1401^{a}	6971 ± 867^{b}	6695 ± 1046^{b}	7057 ± 1213^{b}
K	Root	14478 ± 1892^{a}	11848 ± 1713^{ab}	10049 ± 913^{b}	10006 ± 1460^{b}	10854 ± 1685^{b}
Ca	Leaf	1078 ± 157^{ab}	841 ± 224^{b}	1117 ± 215^{ab}	1148 ± 125^{a}	1163 ± 137^{a}
Ca	Root	1261 ± 282^{a}	755 ± 119^{b}	789 ± 90^{b}	893 ± 330^{ab}	832 ± 217^{b}
Mn	Leaf	219 ± 35^{a}	173 ± 61^{ab}	143 ± 22^{b}	139 ± 19^{b}	151 ± 20^{b}
Mn	Root	89 ± 28^{a}	58 ± 10^{b}	50 ± 6^{b}	43 ± 10^{b}	49 ± 10^{b}
Co	Leaf	0.0057 ± 0.0013^{a}	0.0045 ± 0.0012^{ab}	0.0031 ± 0.0007^{bc}	0.0029 ± 0.0007^{c}	0.0029 ± 0.0003^{c}
Co	Root	0.361 ± 0.072^{a}	0.221 ± 0.039^{b}	0.154 ± 0.022^{b}	0.176 ± 0.081^{b}	0.154 ± 0.038^{b}
Cu	Leaf	2.13 ± 0.22^{a}	1.96 ± 0.28^{ab}	1.57 ± 0.23^{bc}	1.55 ± 0.25^{c}	1.70 ± 0.21^{bc}
Cu	Root	9.3 ± 2.4^{a}	5.0 ± 1.1^{b}	5.0 ± 0.9^{b}	5.9 ± 4.5^{ab}	5.3 ± 1.6^{ab}
Zn	Leaf	9.9 ± 0.9^{a}	8.4 ± 2.0^{ab}	6.7 ± 1.1^{b}	7.1 ± 1.2^{b}	7.2 ± 1.5^{b}
Zn	Root	27.7 ± 4.2^{a}	19.1 ± 3.8^{b}	18.5 ± 2.1^{b}	21.0 ± 6.6^{ab}	18.6 ± 3.9^{b}
As	Leaf	0.025 ± 0.005	0.026 ± 0.004	0.020 ± 0.004	0.026 ± 0.004	0.022 ± 0.003
As	Root	1.31 ± 0.29^{b}	1.04 ± 0.18^{b}	1.50 ± 0.22^{ab}	1.85 ± 0.40^{a}	1.49 ± 0.20^{ab}
Cd	Leaf	0.010 ± 0.004	0.010 ± 0.005	0.007 ± 0.001	0.010 ± 0.005	0.005 ± 0.002
Cd	Root	0.086 ± 0.019^{a}	0.064 ± 0.019^{ab}	0.049 ± 0.007^{b}	0.047 ± 0.010^{b}	0.054 ± 0.009^{b}
Pb	Leaf	0.018 ± 0.004	0.018 ± 0.013	0.023 ± 0.016	0.019 ± 0.004	0.016 ± 0.008
Pb	Root	2.92 ± 0.61^{a}	1.93 ± 0.37^{b}	2.60 ± 0.30^{ab}	2.69 ± 0.80^{ab}	2.35 ± 0.42^{ab}
